# Protein Kinase C Zeta Regulates Human Pancreatic Cancer Cell Transformed Growth and Invasion through a STAT3-Dependent Mechanism

**DOI:** 10.1371/journal.pone.0072061

**Published:** 2013-08-28

**Authors:** Amanda M. Butler, Michele L. Scotti Buzhardt, Shuhua Li, Kristin E. Smith, Alan P. Fields, Nicole R. Murray

**Affiliations:** Department of Cancer Biology, Mayo Clinic, Jacksonville, Florida, United States of America; Indiana University School of Medicine, United States of America

## Abstract

Pancreatic cancer is a very aggressive disease with few therapeutic options. In this study, we investigate the role of protein kinase C zeta (PKCζ) in pancreatic cancer cells. PKCζ has been shown to act as either a tumor suppressor or tumor promoter depending upon the cellular context. We find that PKCζ expression is either maintained or elevated in primary human pancreatic tumors, but is never lost, consistent with PKCζ playing a promotive role in the pancreatic cancer phenotype. Genetic inhibition of PKCζ reduced adherent growth, cell survival and anchorage-independent growth of human pancreatic cancer cells in vitro. Furthermore, PKCζ inhibition reduced orthotopic tumor size in vivo by inhibiting tumor cell proliferation and increasing tumor necrosis. In addition, PKCζ inhibition reduced tumor metastases in vivo, and caused a corresponding reduction in pancreatic cancer cell invasion *in vitro*. Signal transducer and activator of transcription 3 (STAT3) is often constitutively active in pancreatic cancer, and plays an important role in pancreatic cancer cell survival and metastasis. Interestingly, inhibition of PKCζ significantly reduced constitutive STAT3 activation in pancreatic cancer cells *in vitro* and *in vivo*. Pharmacologic inhibition of STAT3 mimicked the phenotype of PKCζ inhibition, and expression of a constitutively active STAT3 construct rescued the transformed phenotype in PKCζ-deficient cells. We conclude that PKCζ is required for pancreatic cancer cell transformed growth and invasion in vitro and tumorigenesis in vivo, and that STAT3 is an important downstream mediator of the pro-carcinogenic effects of PKCζ in pancreatic cancer cells.

## Introduction

Pancreatic cancer is the tenth most commonly diagnosed cancer in the U.S., and ranks fourth in lethality [1]. The overall 5-year survival rate of pancreatic cancer is less than 5% and has not significantly improved over the past 30 years. The lethality of pancreatic cancer is attributed in part to resistance to current chemotherapies [2]. Characterization of novel oncogenic signaling pathways in pancreatic cancer may lead to the identification of more effective therapeutic targets for pancreatic cancer treatment.

Protein Kinase C (PKC) has been implicated in tumorigenesis for over 30 years, since it was first characterized as a receptor for the tumor-promoting phorbol esters [3]. PKC is now known to be a family of related isoforms, and recent studies have characterized the specific roles of individual isoforms in susceptibility to, and development of, cancer [4], [5], [6], [7], [8], [9], [10]. Although members of the atypical PKC (aPKC) sub-family of PKC isoforms are unable to bind and be activated by phorbol esters, their potential role in the cancer phenotype has also been investigated. The two aPKCs, PKC iota (PKCι) and PKC zeta (PKCζ), are structurally similar; however, embryonic knockout of each aPKC reveals unique phenotypes, suggesting non-redundant functions in development and cancer [11], [12]. PKCι promotes cancer development in mouse models of lung and colon cancer, and is an oncogene in lung and ovarian cancer [5], [6], [13], [14], [15]. Similarly, we have demonstrated a pro-carcinogenic role for PKCι in pancreatic cancer cells [16]. In contrast, both tumor promotive and tumor suppressor roles have been attributed to PKCζ [4], [17], [18], however its role in pancreatic cancer has not been evaluated. In the present study, we show that PKCζ is elevated in a subset of human pancreatic tumor tissues compared to matched normal pancreatic epithelium. Furthermore, we demonstrate that inhibition of PKCζ in pancreatic cancer cells significantly impairs the cancer phenotype. Our data also identify STAT3 as an important mediator of PKCζ in the transformed growth and invasion of pancreatic cancer cells.

## Materials and Methods

### Ethics statement

Biospecimens were obtained from the Mayo Clinic Tissue Registry under an approved Mayo Clinic Institutional Review Board protocol. All animal experiments were approved by the Mayo Clinic Institutional Animal Care and Use Committee.

### Patient samples

RNA was isolated from a set of pancreatic adenocarcinoma patient samples for which frozen, paired tumor and non-tumor pancreas tissue was available as described [16]. Hematoxylin and eosin (H&E)-stained sections of matched tumor and adjacent, non-tumor pancreatic tissues were analyzed to confirm the appropriate histology.

### Reagents and cell culture

Human pancreatic cancer cell lines were purchased from American Type Culture Collection and all experiments were performed with cells passaged less than 6 months. Human pancreatic cancer cell lines were maintained in a 5% CO_2_ humidified tissue culture incubator in DMEM with 10% FBS as recommended by American Type Culture Collection. Antibodies were obtained from the following sources: PKCζ, β-actin, phospho-STAT3 (Y705), STAT3, phospho-ERK1/2, ERK1/2 and cleaved caspase-3 (Cell Signaling Technologies), PKCι (BD Transduction Laboratories), 5-bromo-2′-deoxyuridine (BrdUrd) (DakoCytomation) and FLAG (SIGMA Life Sciences).

### RNA isolation and quantitative real-time PCR

Total RNA was isolated using RNAqueous Isolation Kit (Ambion) according to the manufacturer's protocols. TaqMan® Gene Expression Assay primer and probe sets (Applied Biosystems) were used for real-time, quantitative PCR (qPCR) analysis of hGAPDH (Hs99999905_m1), hPKCζ (Hs00177051_m1) and 18S (Hs99999901_s1). qPCR analyses were carried out using 10 ng of cDNA (GAPDH and hPKCζ) or 2 ng cDNA (18S) on an Applied Biosystems 7900 thermal cycler. Data was evaluated using the SDS 2.3 software package. Gene expression in pancreatic tumors and in pancreatic cancer cell lines was normalized to 18S and GAPDH, respectively. All data is expressed as 2^-(*CT*(target)-*CT*(endogenous reference))^.

### Immunohistochemistry and expression analysis

Tissues were processed for immunohistochemical analysis (IHC) as described previously [19]. PKCζ and phospho-STAT3 staining was visualized using the Envision Plus Anti-Rabbit Labeled Polymer-HRP (Dako). Images were captured using Aperio ImageScope and analyzed with Aperio Spectrum software.

### Inhibition of PKCζ expression

Lentiviral vectors expressing short hairpin RNA interference (RNAi) constructs targeting human PKCζ were generated and used to obtain stable transfectants as described previously [20]. PKCζ RNAi #1 construct targets a sequence in the coding region of PKCζ (GTTGTTCCTGGTCATTGAGTA) and PKCζ RNAi #2 construct targets a sequence in the 3′ untranslated region of PKCζ (GACAGACGCTTGCGCCGAGAC). Cell populations carrying the lentiviral constructs were selected and maintained by inclusion of puromycin in the culture media.

### Cell quantitation assay

Cell viability was assessed by MTT assay (CellTiter 96 AQueous One Solution, Promega), as recommended by the manufacturer. Pancreatic cancer cells, Panc-1 (1×10^3^ cells/well) and MiaPaCa-2 (1×10^2^ cells/well) were cultured in a 96-well plate for 1, 3, 5 and 7 days prior to assay.

### Cell death assay

Cell death was assayed using the Cell Death Detection ELISA Plus assay (Roche) according to the manufacturer's protocol.

### Anchorage-independent growth assays

Panc-1 and MiaPaCa-2 cells (5×10^3^) were plated in soft agar and assessed for anchorage-independent growth as described previously [21].

### Orthotopic tumor model

Panc-1 human pancreatic cancer cells (1×10^6^) carrying a retroviral vector encoding firefly luciferase pSIN-Fluc [22] and expressing either NT [16] or PKCζ RNAi were mixed with growth factor reduced Matrigel (Becton Dickinson) and injected into the proximal pancreas of 4–6 week old male athymic nude mice (*n* = 16). All surgeries were performed under isoflurane anesthesia, and mice were administered buprenorphine as an analgesic immediately before and ∼18 hours after the surgery to minimize animal discomfort. Tumor-bearing mice were monitored daily for signs of distress and twice weekly for weight loss. Tumor growth was monitored weekly by fluorescence imaging. Briefly, mice were injected intraperitoneally with D-Luciferin solution (Xenogen) at a dose of 150 mg/kg body weight, anesthetized with isoflurane and imaged using a bioluminescence imaging system (Caliper Life Sciences-Xenogen, Hopkinton, MA). One hour prior to sacrifice, mice were injected intraperitoneally with 100 mg/kg BrdUrd.

### Orthotopic tumor analysis

Tumors from mice injected with PKCζ RNAi cells were formalin-fixed and analyzed for proliferation (BrdUrd incorporation) by immunohistochemistry (IHC), as previously described for NT RNAi tumors [16]. Apoptosis was assessed by detection of caspase-3 cleavage as described previously [19], [23]. Tumor necrosis was identified in H&E stained tissue. Spectrum software was used to calculate percent necrotic tumor area by dividing necrotic tumor area by total tumor area. Tumor metastases were identified by gross anatomical evaluation of abdominal and chest organs upon completion of the study, and verified by H&E staining of the metastatic lesions as described for NT RNAi tumors [16].

### Cellular invasion assay

Cellular invasion was assayed using matrigel-coated invasion chambers (BD Biosciences) according to the manufacturer's protocol. Briefly, 5×10^4^ human pancreatic cancer cells were plated in serum-free media in the top chamber, and DMEM containing 2.5% FBS was used as the chemoattractant in the bottom chamber. Cells were allowed to invade for 24 hrs at 37°C and cells were then fixed, stained and quantitated as previously described [20].

### Expression of constitutively active STAT3 (STAT3-C)

Cells were infected with Adeno-Null or FLAG tagged-Adeno-STAT3-C [24]. Protein expression was determined by immunoblot analysis of total cell lysates. Immunoblot analysis was performed on cells isolated at 60–80% confluence.

### Statistical analysis

Two-way ANOVA and Student *t*-test were used to evaluate the statistical significance of the results. *p*<0.05 was considered statistically significant.

## Results

### PKCζ is elevated in a subset of human pancreatic tumors

**Figure 1:**
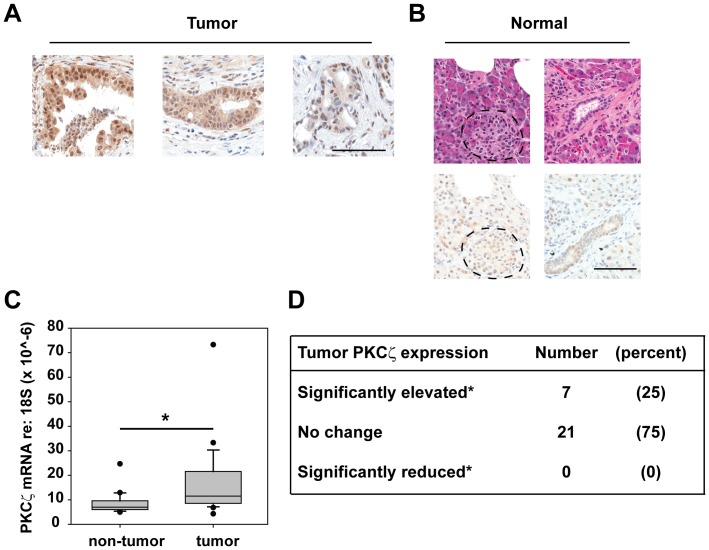
PKCζ is elevated in a subset of human pancreatic tumors. A and B) IHC detection of PKCζ expression in representative human pancreatic tumors (A; Tumor) and adjacent non-tumor tissue (B; Normal). B) Serial sections stained with H&E are provided to distinguish acinar, islet (top left image, pancreatic islet is outlined) and ductal cells (top right image). All images in the same panel are the same magnification. Bars = 100 µm. C) Quantitative PCR analysis of PKCζ mRNA expression was performed on 28 matched patient pancreatic adenocarcinoma and non-tumor samples. PKCζ expression was normalized to 18S abundance; *p = 0.001 calculated by paired *t*-test. D) PKCζ expression is significantly elevated in a subset of pancreatic tumors. PKCζ was overexpressed in 25% of pancreatic tumors analyzed, as defined by tumor mRNA abundance greater than 2 standard deviations above the average of PKCζ mRNA abundance in all adjacent non-tumor pancreas samples.

We began our study by evaluating PKCζ expression in primary human pancreatic tumors and surrounding non-tumor tissue (Figure 1). Clinical and demographic information for this patient population is published [16]. Immunohistochemical detection of PKCζ protein in representative pancreatic tumor tissues revealed a variable level of PKCζ expression which localized to both the nucleus and cytoplasm (Figure 1A). PKCζ expression was also detected at a variable but lower level in non-tumor, pancreatic cell types (Figure 1B). Islet and acinar cells of the non-tumor pancreas showed low PKCζ expression (Figure 1B, left panels). The expression of PKCζ in ductal cells was similar to, or slightly higher than, the expression in islet and acinar cells (Figure 1B, right panels). We next evaluated PKCζ mRNA expression in a panel of 28 paired human pancreatic adenocarcinoma and adjacent, non-tumor pancreas. PKCζ mRNA expression was detected in all 28 primary pancreatic tumors analyzed (data not shown). Analysis of paired samples revealed that PKCζ expression was significantly higher in tumors than in paired, non-tumor tissue (Figure 1C). PKCζ mRNA expression was significantly elevated in 25% of pancreatic tumors, compared to the average PKCζ mRNA expression in non-tumor pancreas, and no tumors exhibited a significant reduction in PKCζ mRNA expression (Figure 1D). Analysis of the relationship between PKCζ mRNA expression and patient survival was conducted, but in this small cohort no correlation was observed.

### PKCζ regulates the transformed phenotype of pancreatic cancer cells in vitro

**Figure 2:**
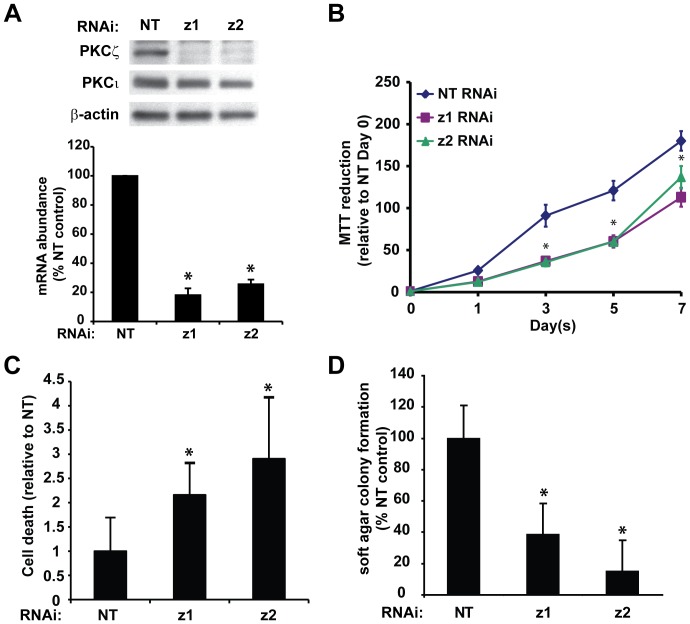
Inhibition of PKCζ expression reduces survival and transformed growth of pancreatic cancer cells. Panc-1 cells stably carrying lentivirus expressing either control, non-targeting (NT) or PKCζ-targeting RNAi (z1 and z2) were assessed for A) PKCζ and PKCι protein expression by immunoblot analysis (top), and PKCζ mRNA abundance by qPCR analysis (bottom); B) cell viability (MTT colorimetric assay); C) cellular death (detected by Cell Death Detection ELISA) and D) anchorage-independent growth (colony formation in soft agar). For each panel Bars = average of 3 or more replicates±SD and graph is representative of 3 or more independent experiments. *p<0.05 vs NT.

To directly assess the role of PKCζ in the pancreatic cancer phenotype, we used two different RNAi constructs to inhibit PKCζ expression in two well-characterized human pancreatic cancer cell lines, Panc-1 (Figure 2A) and MiaPaCa-2 (Figure S1A). Stably selected cell populations consistently exhibited 70% or greater inhibition of PKCζ mRNA expression, with a corresponding decrease in PKCζ protein expression (Figure 2A and S1A). Selectivity of the PKCζ-targeted RNAi constructs is confirmed by the lack of effect of these constructs on the expression of the closely related atypical PKCι isozyme (Figure 2A and S1A). Inhibition of PKCζ expression resulted in a small but significant decrease in log-phase, adherent cell growth (Figure 2B and S1B) and an increase in basal cell death (Figure 2C). Furthermore, PKCζ knock down (KD) significantly decreased pancreatic cancer cell anchorage-independent growth (soft agar colony formation) (Figure 2D and S1C), indicating that PKCζ is critical for pancreatic cancer cell survival and the transformed phenotype.

### PKCζ plays a critical role in pancreatic tumorigenesis

**Figure 3:**
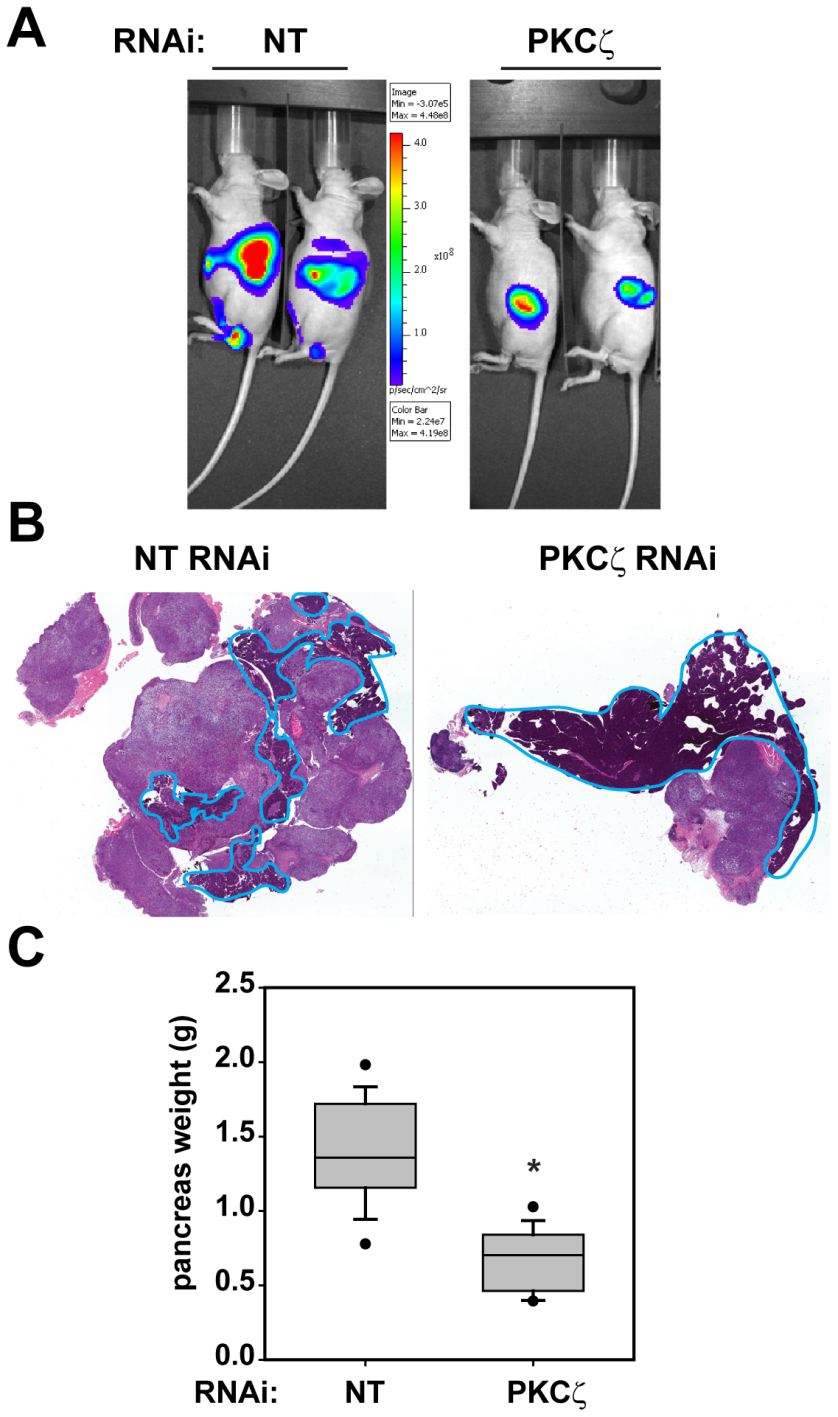
Inhibition of PKCζ expression significantly reduces orthotopic pancreatic tumor size. A) Representative bioluminescent imaging of mice with orthotopic Panc-1 NT and PKCζ RNAi pancreatic tumors. B) Representative H&E stained sections of orthotopic Panc-1 NT and PKCζ RNAi pancreatic tumors. The remaining normal mouse pancreas is circled in blue. C) Inhibition of PKCζ significantly decreased the pancreas and orthotopic tumor weight; n = 16; *p<0.001.

**Figure 4:**
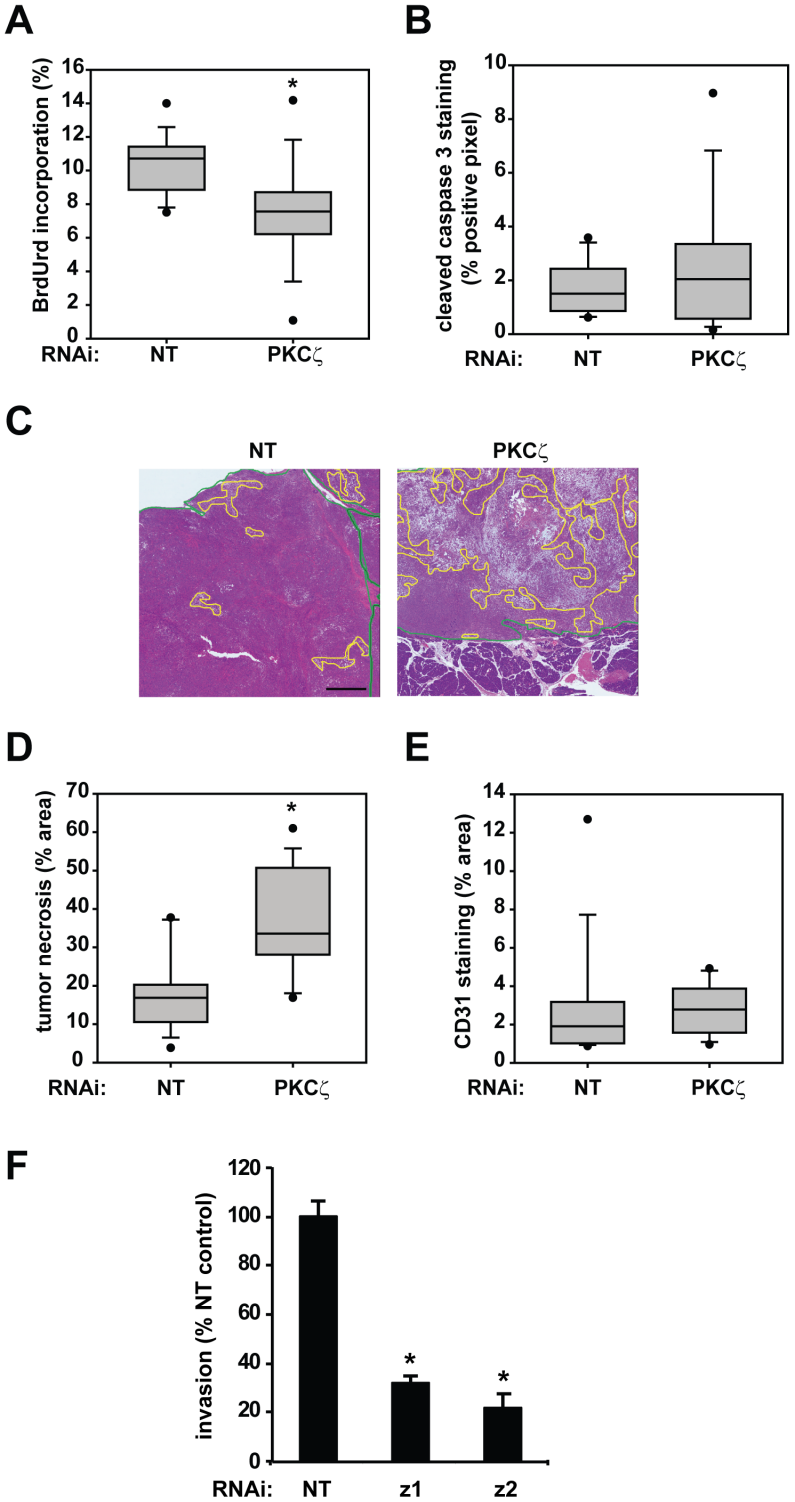
Inhibition of PKCζ expression significantly reduces orthotopic pancreatic tumor proliferation and increases tumor necrosis. A) Quantitative analysis of tumor proliferation detected by BrdUrd incorporation; *p<0.003. B) Quantitative analysis of tumor apoptosis detected by cleaved caspase-3 staining. C) Representative H&E stained orthotopic Panc-1 NT and PKCζ RNAi pancreatic tumors with areas of necrosis identified (yellow outline) (bar = 1 mm). Green line delineates tumor tissue. D) Quantitative analysis of tumor necrosis plotted as percent of total tumor area; *p<0.0002. E) Quantitative analysis of tumor vascularity, as determined by percent area CD31 staining. A–E) *n* = 16 NT RNAi tumors and 15 PKCζ RNAi tumors. F) Panc-1 NT and PKCζ RNAi cells (z1 and z2) were assessed for cellular invasion through Matrigel-coated chambers. Bars = average of 3 or more replicates+/−SD and graph is representative of 2 or more independent experiments. *p<0.05 vs NT.

We next investigated the effect of PKCζ KD on pancreatic tumor formation and growth using a previously described Panc-1 orthotopic tumor model [16]. Panc-1 cells expressing the firefly luciferase gene (pSIN-Fluc) and either NT or PKCζ RNAi were injected into the pancreas of nude mice to form orthotopic tumors. Tumor growth was monitored by bioluminescence detection (Figure 3A), and mice were harvested 5 weeks after inoculation. Tumor formation was observed in all mice injected with Panc-1 cells expressing either RNAi construct; however, final pancreas weight was significantly lower in mice bearing PKCζ RNAi tumors, due to reduced tumor size (Figure 3B and 3C). We hypothesized that, similar to the effect of PKCζ KD in vitro (Figure 2B–D), the reduced tumor size of PKCζ KD Panc-1 cells in vivo was due to reduced tumor cell proliferation and enhanced tumor cell death. The level of BrdUrd incorporation, a measure of tumor proliferation, was evaluated in Panc-1 PKCζ RNAi tumors and compared to the level of BrdUrd incorporation in Panc-1 NT RNAi tumors [16]. As predicted, tumor proliferation was significantly reduced in PKCζ RNAi tumors compared to NT RNAi tumors (Figure 4A). Interestingly, we did not observe a significant effect of PKCζ KD on tumor apoptosis, detected by cleaved caspase-3 (Figure 4B). However, PKCζ RNAi tumors had a significantly higher level of necrosis than NT RNAi tumors (Figure 4C and 4D). Tumor necrosis results from an accumulation of tumor cell death, which can occur when a tumor outgrows its blood supply. Although PKCζ RNAi tumors are drastically smaller than NT RNAi tumors, they do not exhibit a decrease in tumor blood vessel density as quantified by CD31 staining (Figure 4E). These data suggest that the reduced tumor volume of PKCζ RNAi pancreatic tumors is the result of the cumulative effect of decreased cell proliferation and survival over the time course of the in vivo experiment.

### PKCζ plays an important role in pancreatic cancer cell invasion

In the pancreatic orthotopic tumor model, Panc-1 cells form both primary tumors and metastatic lesions [16]. Metastases to the kidney, liver, diaphragm, and mesentery were observed in more than 50% of the mice harboring NT RNAi tumors (Table 1, [16]). In contrast, no tumor metastasis to the mesentery or diaphragm was identified in mice carrying PKCζ RNAi tumors; only 2 of 15 PKCζ RNAi tumor-bearing mice (13%) had metastases to their kidneys, and only 1 of 15 PKCζ RNAi tumor-bearing mice (6%) had a liver metastasis (Table 1). These data are consistent with an inhibitory effect of PKCζ KD on pancreatic tumor metastasis. However, we cannot rule out the possibility that the decreased metastasis observed in PKCζ RNAi tumors may be secondary to the significantly reduced size of the tumors. If PKCζ regulates tumor metastasis in vivo, it is likely to also regulate aspects of the metastatic phenotype, such as cellular invasion, in vitro. Indeed, cellular invasion was significantly decreased in PKCζ RNAi cells, when compared to NT RNAi pancreatic cancer cells (Figure 4F and S2). These results demonstrate a role for PKCζ in pancreatic cancer cell invasion, and are consistent with a role for PKCζ in the metastatic phenotype of pancreatic cancer cells in vivo.

**Table 1:** PKCζ inhibition reduces orthotopic pancreatic tumor metastasis.

Site of metastasis	% metastasis		*p* value
	NT RNAi	PKCζ RNAi	
Liver	56	6	0.006
Kidney	75	13	0.001
Mesentery	63	0	0.0002
Diaphragm	63	0	0.0002

### PKCζ regulates STAT3 activation

**Figure 5:**
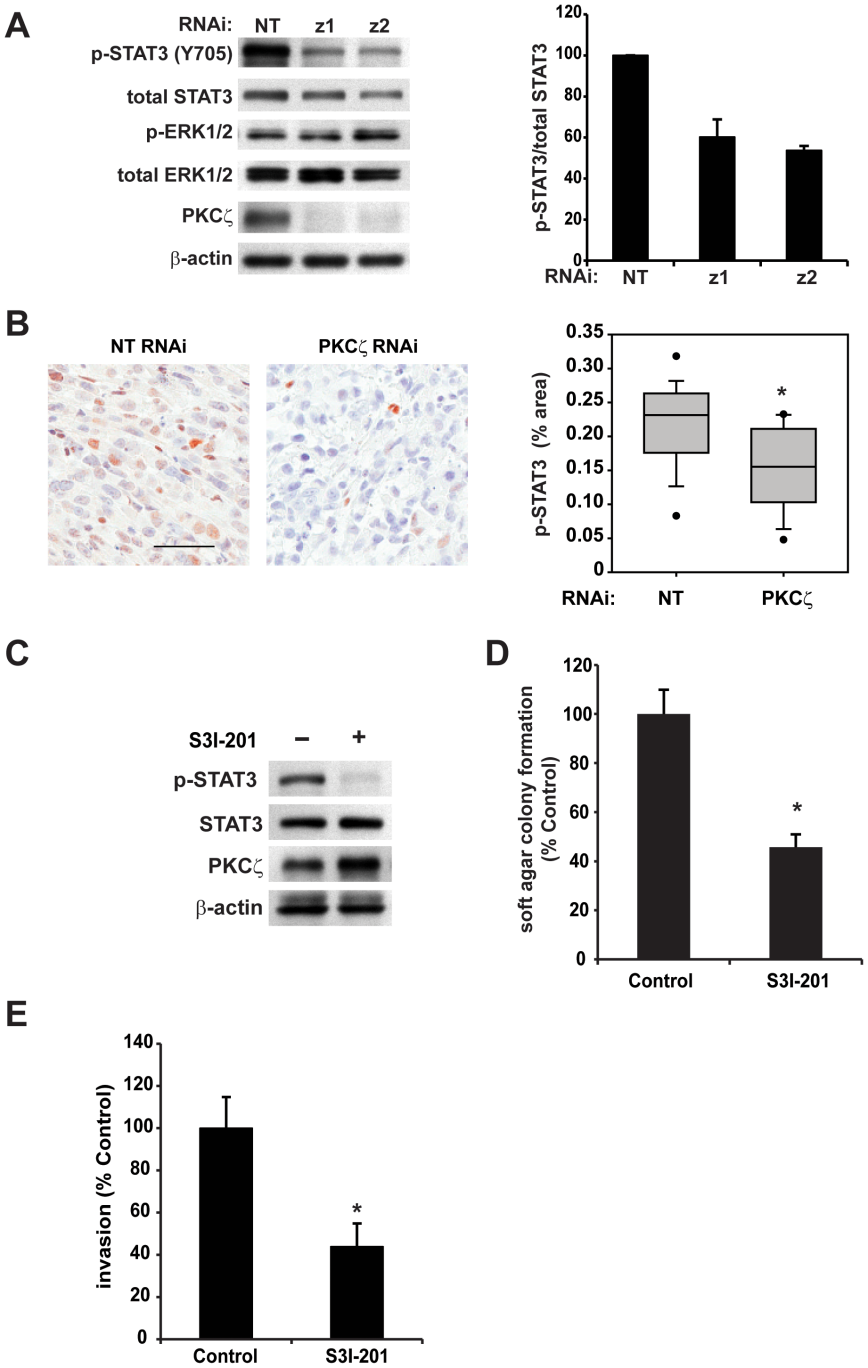
PKCζ expression regulates STAT3 phosphorylation. A) Inhibition of PKCζ expression decreases constitutive STAT3 activation (detected as phospho-STAT3 Y705) but not ERK1/2 activation (detected as phospho-ERK1/2). Immunoblot analysis was performed on total cell lysates from Panc-1 NT and PKCζ RNAi cells (left) and expression analysis of immunoblot detection was performed (right) *n* = 3. B) Representative IHC detection of p-STAT3 in orthotopic Panc-1 NT and PKCζ RNAi pancreatic tumors (left), bar = 50 µm. Quantitative analysis of pSTAT3 IHC staining (right). C–E) The effect of STAT3 inhibitor (S3I-201) on C) STAT3 phosphorylation, D) anchorage-independent growth in soft agar and E) cellular invasion through Matrigel-coated chambers. In all assays, S3I-201 was used at 100 µm and an equal volume DMSO used as control diluent. For invasion assay, cells were pre-treated with S3I-201 or DMSO for 48 hours prior to initiation of the assay. Bars = average of 3 or more replicates+/−SD, and graph is representative of 2 or more independent experiments. *p<0.05.

Signal transducer and activator of transcription-3 (STAT3) is a transcription factor that integrates numerous extracellular signals to regulate cancer-promoting cellular processes [25], [26]. Constitutive STAT3 activation is a hallmark of many human cancers, including pancreatic cancer [27]. STAT3 activation promotes the oncogenic phenotype of pancreatic cancer, and loss of STAT3 prevents pancreatic cancer development and progression in a mouse model of *Kras*-mediated pancreatic cancer [27], [28]. Furthermore, inhibition of STAT3 activity in pancreatic cancer cells also reduces cell survival, invasion and tumor growth [28], [29]. Given the striking similarity between the reported phenotype of STAT3 inhibition and the phenotype we observed with inhibition of PKCζ, we asked whether PKCζ expression regulates STAT3 activity in pancreatic cancer cell lines. A significant reduction in STAT3 activation, detected as phosphorylation of STAT3 on Tyr705, was observed in pancreatic cancer cells expressing PKCζ RNAi (Figures 5A and S3A). Furthermore, STAT3 activation was significantly reduced in PKCζ RNAi tumors when compared to NT RNAi tumors (Figure 5B), indicating that PKCζ regulates STAT3 activation in pancreatic cancer cells both in vitro and in vivo. Since PKCζ has also been implicated in the regulation of ERK1/2 activation in cancer and non-cancer cell types [30], [31], [32], [33], we analyzed the effect of PKCζ RNAi on ERK1/2 phosphorylation in human pancreatic cancer cells. Unlike STAT3 phosphorylation, ERK1/2 phosphorylation was not altered by a significant reduction in PKCζ expression (Figures 5A and S3A) suggesting that PKCζ expression does not regulate signaling through the ERK1/2 signaling pathway.

### STAT3 inhibition reduces the transformed phenotype of pancreatic cancer cells

To determine whether reduced STAT3 activation may be responsible for some of the effects of PKCζ KD, we assessed the effect of a pharmacological inhibitor of STAT3 on the transformed phenotype of pancreatic cancer cells. Treatment of pancreatic cancer cells with S3I-201, a small molecule that disrupts STAT3 SH2-phospho-tyrosine interactions [34], reduced STAT3 activation (Figure 5C and S3B) and significantly reduced anchorage-independent growth (Figures 5D) and cellular invasion (Figure 5E and S3C), similar to the effect of PKCζ inhibition. Taken together, these data demonstrate that inhibition of PKCζ expression reduces STAT3 activity in pancreatic cancer cells, and that PKCζ expression and STAT3 activity positively regulate pancreatic cancer cell transformed growth and invasion.

### Constitutively active STAT3 can reconstitute the transformed phenotype in PKCζ RNAi pancreatic cancer cells

**Figure 6:**
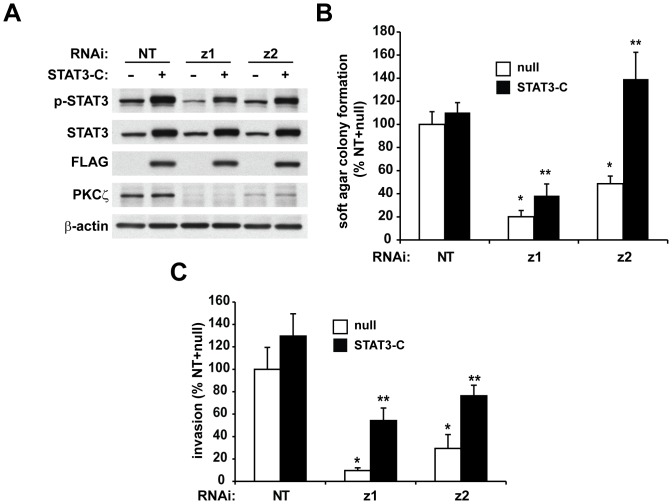
Constitutively active STAT3 rescues the transformed phenotype in PKCζ RNAi-expressing cells. Panc-1 cells expressing NT or PKCζ RNAi were infected with adenoviral constructs expressing either null (control), or constitutively active, FLAG-tagged STAT3 (STAT3-C). A) Immunoblot analysis of p-STAT3, STAT3, FLAG, PKCζ and β-actin expression. Cells were assessed for B) anchorage-independent growth in soft agar and C) cellular invasion through Matrigel-coated chambers. For each graph: Bars = average of 3 or more replicates±SD and graph is representative of 2 or more independent experiments. *significantly reduced compared to NT/null, p<0.05; **significantly increased compared to null-treated, p<0.05.

To test the hypothesis that STAT3 is a critical downstream effector of PKCζ in pancreatic cancer cells, we assessed whether expression of a constitutively active STAT3 construct (STAT3-C) could rescue the effects of PKCζ inhibition in Panc-1 cells. Panc-1 NT and PKCζ RNAi cells were infected with adenovirus expressing flag-tagged, STAT3-C or control (null) adenovirus (Figure 6A). Expression of STAT3-C significantly recovered anchorage-independent growth of Panc-1 PKCζ RNAi cells, without significantly affecting the anchorage-independent growth of NT RNAi cells (Figure 6B). In addition, the reduced cellular invasion phenotype of PKCζ RNAi cells was significantly recovered by expression of STAT3-C (Figure 6C). Taken together, these data demonstrate that increased cellular STAT3 activity can rescue the anti-oncogenic phenotype of PKCζ RNAi cells, and demonstrate that PKCζ mediates pancreatic cancer cell transformation, at least in part, through regulation of STAT3 activity.

## Discussion

Functional studies have shown that the role of PKCζ in regulating the cancer phenotype varies by tumor type, model system and stage of disease. For example, inhibition of PKCζ expression in a colon cancer cell line reduces proliferation in vitro and tumor size in vivo; however, genetic inhibition of PKCζ in mouse intestinal epithelium does not affect tumorigenesis in the APCmin/+ mouse model of intestinal cancer initiation and progression [7], [35]. In contrast, genetic inhibition of PKCζ in a mouse model of *Kras^G12D^*-induced lung tumorigenesis reveals a tumor suppressor role [4], while inhibition of PKCζ expression in lung cancer cells has no effect on transformed growth in vitro [20]. In the present study, we evaluated the specific role of PKCζ in the biology of pancreatic cancer cells, using PKC isotype-specific RNAi to inhibit PKCζ expression. We demonstrate that PKCζ KD reduced pancreatic cancer cell proliferation and cell survival in vitro. We further show that PKCζ KD in pancreatic cancer cells significantly reduced transformed growth in vitro, corresponding to a significant reduction in tumor size in vivo. These data strongly suggest that PKCζ is required for maintenance of the transformed phenotype of pancreatic cancer cells.

PKCζ has been implicated in the invasive phenotype of human cancers [36], [37], [38]. RNAi-mediated, specific inhibition of PKCζ reduces breast cancer and glioblastoma cell invasion in vitro [37], [38] and reduces prostate cancer cell invasion in vitro and in vivo [36]. Interestingly, each of these reports attributes PKCζ to a distinct invasive signaling pathway, suggesting a broad role for PKCζ in cancer cell invasion [36], [37], [38]. Consistent with the phenotype observed in other cancers, we determined that inhibition of PKCζ expression not only inhibited the transformed growth of pancreatic cancer cells, but also repressed their invasive potential in vitro. Furthermore, PKCζ KD significantly reduced pancreatic tumor metastasis, indicating that PKCζ regulates pancreatic tumor cell invasion in vivo, as well as in vitro.

The prognostic value of PKCζ expression in cancer is not well documented. However, several recent reports have implicated PKCζ as a predictor of poor outcome for cancer patients. High PKCζ predicts poor disease-specific survival of patients with soft tissue sarcoma [39]. Likewise, PKCζ is elevated in prostate cancer, and high PKCζ expression predicts poor survival of prostate cancer patients [36]. We evaluated the expression of PKCζ in pancreatic cancer, and determined that PKCζ was clearly elevated in a sub-set of pancreatic cancers. However, our small sample size coupled with the poor overall prognosis of pancreatic cancer patients precluded determination of a potential prognostic role of PKCζ expression. Ongoing tissue collections will facilitate future investigation of the ability of PKCζ expression to predict outcome in a larger cohort of pancreatic cancer patients.

While PKCζ expression has been recently characterized to be elevated and predict poor survival in several cancers [36], [39], little is known about the regulation of PKCζ expression. However, PKCζ has been shown to be activated by several signaling pathways known to promote oncogenic signaling in pancreatic cancer. Phosphatidylinositol-3,4-5-trisphosphate (Ptdins-3,4,5-P3), the product of phosphoinositide 3-kinase, can directly bind and activate PKCζ, and also activates Ptdins-3,4,5-P3-activated phosphoinositide-dependent kinase 1-mediated phosphorylation and activation of PKCζ [40], [41], [42]. In head and neck squamous carcinoma cells, PKCζ is tyrosine phosphorylated and activated by epidermal growth factor receptor [31]. Future studies will investigate whether either of these pathways, both frequently dysregulated in pancreatic cancer, modulates PKCζ signaling in pancreatic cancer cell lines.

In contrast to our observation that inhibition of PKCζ repressed pancreatic tumor growth and metastasis, genetic inhibition of PKCζ in *Kras^G12D^*-induced lung tumors promotes tumor growth and progression [4]. The tumor suppressive role of PKCζ in K-ras-mediated lung tumorigenesis is mediated by repression of STAT3 activation in the tumor cells [4]. Interestingly, STAT3 is often constitutively activated in pancreatic tumors and pancreatic cancer cell lines [43], [44], and activated STAT3 promotes pancreatic cancer cell survival, transformed growth, invasion, and tumor metastasis [27], [28], [45]. Consistent with an oncogenic role in pancreatic cancer, we show that inhibition of STAT3 reduced pancreatic cancer cell invasion and soft agar colony formation, similar to the effect of PKCζ inhibition. Furthermore, inhibition of PKCζ expression significantly reduced constitutive STAT3 phosphorylation in pancreatic cancer cells grown in culture, and as orthotopic tumors. In contrast, inhibition of STAT3 had no effect on PKCζ expression, suggesting that PKCζ positively regulates constitutive STAT3 activity in pancreatic cancer. In support of this hypothesis, expression of a constitutively active STAT3 construct was able to significantly overcome the inhibition of the transformed phenotype in PKCζ RNAi cells, without affecting PKCζ expression. Therefore, one mechanism by which PKCζ expression positively regulates the oncogenic phenotype of pancreatic cancer cells is by promoting constitutive STAT3 activity. While the opposing roles of PKCζ in both tumorigenesis and STAT3 activation in pancreas and lung may be explained by differences in the tissue type, they may also be due to cancer-specific roles for PKCζ in tumor initiation and maintenance. Analysis of the role of PKCζ in the initiation and progression of pancreatic cancer will require the use of a genetic (*Kras^G12D^*-induced) mouse model of pancreatic tumor formation.Resistance to chemotherapy is a primary characteristic of pancreatic cancer that contributes to the high lethality of this disease. Constitutive STAT3 signaling not only promotes tumor growth and metastasis, but is also associated with chemotherapeutic resistance of cancer cells [46], [47]. While inhibition of Src or EGFR signaling pathways temporarily reduces constitutive STAT3 in pancreatic cancer cell lines, reactivation of STAT3 occurs rapidly [46], [47]. Inhibition of STAT3 sensitizes pancreatic cancer cells to tumor growth inhibition and apoptosis induced by Src or EGFR inhibitors, suggesting that co-inhibition of STAT3 may increase the efficacy of targeted therapeutics [46], [47]. However, currently no clinically relevant inhibitors of STAT3 are available for use in patients [48]. Our observation that inhibition of PKCζ expression significantly and stably reduced STAT3 activation in pancreatic cancer cells suggests that PKCζ inhibition may be a means to stably suppress STAT3 activity, and thereby enhance the sensitivity of pancreatic cancer cells to current chemotherapies. An isotype-selective inhibitor of PKCζ has recently been described [49], [50]. Based on the results of this study, an evaluation of the effect of pharmacological inhibition of PKCζ on pancreatic cancer cell transformed growth, invasion and chemoresistance is clearly warranted.

In the present study, we demonstrate that inhibition of PKCζ decreases pancreatic cancer cell transformed growth, invasion and migration in vitro, and tumor growth in vivo. We provide strong evidence that PKCζ RNAi-mediated reduction in invasion and soft agar colony formation is due, at least in part, to down-regulation of constitutive STAT3 activity in pancreatic cancer cells. This is the first report to document a cancer promotive role for PKCζ in pancreatic cancer, and to implicate PKCζ in the positive regulation of constitutive STAT3 signaling in cancer cells. Our future studies will investigate the mechanism by which PKCζ promotes STAT3 activation in pancreatic cancer cells.

## Supporting Information

Figure S1 **Inhibition of PKCζ expression reduces survival and transformed growth of MiaPaca-2 pancreatic cancer cells.** MiaPaca-2 cells stably carrying lentiviral constructs expressing either control, non-targeting (NT), or PKCζ-targeting RNAi (z1 and z2) were assessed for A) PKCζand PKCι protein expression by immunoblot analysis (top), and PKCζ mRNA abundance by qPCR analysis (bottom); B) cell viability (MTT colorimetric assay); C) anchorage-independent growth (colony formation in soft agar). PKCζ RNAi #3 (z3) construct targets a sequence in the coding region of PKCζ (CATGAAAGTGGTGAAGAAAGA). For each panel Bars = average of 3 or more replicates±SD and graph is representative of 2 or more independent experiments. *p<0.05 vs NT.
(TIF)

Figure S2 **PKCζ expression regulates MiaPaca-2 cell invasion.** MiaPaca-2 NT and PKCζ RNAi cells were assessed for cellular invasion through Matrigel-coated chambers. Bars = average of 3 or more replicates+/−SD; graph is representative of 2 or more independent experiments. *p<0.05 vs NT.
(EPS)

Figure S3 **PKCζ expression regulates STAT3 activation in MiaPaca-2 cells.** A) Inhibition of PKCζ expression decreases constitutive STAT3 activation (p-STAT3) but has no effect on ERK1/2 phosphorylation (p-ERK). Immunoblot analysis was performed on total cell lysates from MiaPaca-2 NT and PKCζ RNAi cells. B) Inhibition of STAT3 (S3I-201) decreases p-STAT3. Immunoblot analysis was performed on total cell lysates from MiaPaca-2 NT and PKCζ RNAi cells. C) S3I-201 significantly reduces MiaPaca-2 cell invasion. Bars = average of 3 or more replicates±SD and graph is representative of 2 or more independent experiments. *p<0.05 vs Control. For all panels S3I-201 was used at 100 µm with DMSO as control diluent.
(TIF)

## References

[1] Siegel R, Naishadham D, Jemal A (2012) Cancer statistics, 2012. CA Cancer J Clin 62: 10–29. 22237781 10.3322/caac.20138

[2] Lebedeva IV, Sarkar D, Su ZZ, Gopalkrishnan RV, Athar M, et al (2006) Molecular target-based therapy of pancreatic cancer. Cancer Res 66: 2403–2413. 16489047 10.1158/0008-5472.CAN-05-3510

[3] Castagna M, Takai Y, Kaibuchi K, Sano K, Kikkawa U, et al (1982) Direct activation of calcium-activated, phospholipid-dependent protein kinase by tumor-promoting phorbol esters. J Biol Chem 257: 7847–7851. 7085651

[4] Galvez AS, Duran A, Linares JF, Pathrose P, Castilla EA, et al (2009) Protein kinase Cζ represses the interleukin-6 promoter and impairs tumorigenesis in vivo. Molecular and Cellular Biology 29: 104–115. 18955501 10.1128/MCB.01294-08 PMC2612492

[5] Murray NR, Jamieson L, Yu W, Zhang J, Gokmen-Polar Y, et al (2004) Protein kinase C{iota} is required for Ras transformation and colon carcinogenesis in vivo. J Cell Biol 164: 797–802. 15024028 10.1083/jcb.200311011 PMC2172278

[6] Murray NR, Weems J, Braun U, Leitges M, Fields AP (2009) Protein kinase C betaII and PKCiota/lambda: collaborating partners in colon cancer promotion and progression. Cancer Res 69: 656–662. 19147581 10.1158/0008-5472.CAN-08-3001 PMC2688739

[7] Oster H, Leitges M (2006) Protein kinase C alpha but not PKCzeta suppresses intestinal tumor formation in ApcMin/+ mice. Cancer Res 66: 6955–6963. 16849539 10.1158/0008-5472.CAN-06-0268

[8] Caino MC, Lopez-Haber C, Kissil JL, Kazanietz MG (2012) Non-small cell lung carcinoma cell motility, rac activation and metastatic dissemination are mediated by protein kinase C epsilon. PLoS ONE 7: e31714. 22384062 10.1371/journal.pone.0031714 PMC3288050

[9] Gonzalez-Guerrico AM, Meshki J, Xiao L, Benavides F, Conti CJ, et al (2005) Molecular mechanisms of protein kinase C-induced apoptosis in prostate cancer cells. J Biochem Mol Biol 38: 639–645. 16336777 10.5483/bmbrep.2005.38.6.639

[10] Fields AP, Murray NR (2008) Protein kinase C isozymes as therapeutic targets for treatment of human cancers. Adv Enzyme Regul 48: 166–178. 18167314 10.1016/j.advenzreg.2007.11.014 PMC2586109

[11] Leitges M, Sanz L, Martin P, Duran A, Braun U, et al (2001) Targeted disruption of the zetaPKC gene results in the impairment of the NF-kappaB pathway. Mol Cell 8: 771–780. 11684013 10.1016/s1097-2765(01)00361-6

[12] Soloff RS, Katayama C, Lin MY, Feramisco JR, Hedrick SM (2004) Targeted deletion of protein kinase C lambda reveals a distribution of functions between the two atypical protein kinase C isoforms. J Immunol 173: 3250–3260. 15322187 10.4049/jimmunol.173.5.3250

[13] Regala RP, Davis RK, Kunz A, Khoor A, Leitges M, et al (2009) Atypical protein kinase Cι is required for bronchioalveolar stem cell expansion and lung tumorigenesis. Cancer Research 69: 7603–7611. 19738040 10.1158/0008-5472.CAN-09-2066 PMC2756303

[14] Regala RP, Weems C, Jamieson L, Khoor A, Edell ES, et al (2005) Atypical protein kinase C iota is an oncogene in human non-small cell lung cancer. Cancer Res 65: 8905–8911. 16204062 10.1158/0008-5472.CAN-05-2372

[15] Eder AM, Sui X, Rosen DG, Nolden LK, Cheng KW, et al (2005) Atypical PKCiota contributes to poor prognosis through loss of apical-basal polarity and cyclin E overexpression in ovarian cancer. Proc Natl Acad Sci U S A 102: 12519–12524. 16116079 10.1073/pnas.0505641102 PMC1188258

[16] Scotti ML, Bamlet WR, Smyrk TC, Fields AP, Murray NR (2010) Protein kinase Cι is required for pancreatic cancer cell transformed growth and tumorigenesis. Cancer Research 70: 2064–2074. 20179210 10.1158/0008-5472.CAN-09-2684 PMC2881466

[17] Nazarenko I, Jenny M, Keil J, Gieseler C, Weisshaupt K, et al (2010) Atypical protein kinase C zeta exhibits a proapoptotic function in ovarian cancer. Mol Cancer Res 8: 919–934. 20501645 10.1158/1541-7786.MCR-09-0358

[18] Mustafi R, Cerda S, Chumsangsri A, Fichera A, Bissonnette M (2006) Protein Kinase-zeta inhibits collagen I-dependent and anchorage-independent growth and enhances apoptosis of human Caco-2 cells. Mol Cancer Res 4: 683–694. 16940160 10.1158/1541-7786.MCR-06-0057

[19] Calcagno SR, Li S, Colon M, Kreinest PA, Thompson EA, et al (2008) Oncogenic K-ras promotes early carcinogenesis in the mouse proximal colon. Int J Cancer 122: 2462–2470. 18271008 10.1002/ijc.23383 PMC3908548

[20] Frederick LA, Matthews JA, Jamieson L, Justilien V, Thompson EA, et al (2008) Matrix metalloproteinase-10 is a critical effector of protein kinase Cι-Par6α-mediated lung cancer. Oncogene 27: 4841–4853. 18427549 10.1038/onc.2008.119 PMC2750877

[21] Regala RP, Weems C, Jamieson L, Copland JA, Thompson EA, et al (2005) Atypical protein kinase Ciota plays a critical role in human lung cancer cell growth and tumorigenicity. J Biol Chem 280: 31109–31115. 15994303 10.1074/jbc.M505402200

[22] Hasegawa K, Nakamura T, Harvey M, Ikeda Y, Oberg A, et al (2006) The use of a tropism-modified measles virus in folate receptor-targeted virotherapy of ovarian cancer. Clin Cancer Res 12: 6170–6178. 17062694 10.1158/1078-0432.CCR-06-0992

[23] Calcagno SR, Li S, Shahid MW, Wallace MB, Leitges M, et al (2011) Protein kinase C iota in the intestinal epithelium protects against dextran sodium sulfate-induced colitis. Inflamm Bowel Dis 17: 1685–1697. 21744423 10.1002/ibd.21547 PMC3116999

[24] Haga S, Terui K, Zhang HQ, Enosawa S, Ogawa W, et al (2003) Stat3 protects against Fas-induced liver injury by redox-dependent and -independent mechanisms. J Clin Invest 112: 989–998. 14523036 10.1172/JCI17970 PMC198521

[25] Frank DA (2007) STAT3 as a central mediator of neoplastic cellular transformation. Cancer Lett 251: 199–210. 17129668 10.1016/j.canlet.2006.10.017

[26] Turkson J, Jove R (2000) STAT proteins: novel molecular targets for cancer drug discovery. Oncogene 19: 6613–6626. 11426647 10.1038/sj.onc.1204086

[27] Lesina M, Kurkowski MU, Ludes K, Rose-John S, Treiber M, et al (2011) Stat3/Socs3 activation by IL-6 transsignaling promotes progression of pancreatic intraepithelial neoplasia and development of pancreatic cancer. Cancer Cell 19: 456–469. 21481788 10.1016/j.ccr.2011.03.009

[28] Corcoran RB, Contino G, Deshpande V, Tzatsos A, Conrad C, et al (2011) STAT3 plays a critical role in KRAS-induced pancreatic tumorigenesis. Cancer Res 71: 5020–5029. 21586612 10.1158/0008-5472.CAN-11-0908 PMC3693754

[29] Zhang X, Yue P, Page BD, Li T, Zhao W, et al (2012) Orally bioavailable small-molecule inhibitor of transcription factor Stat3 regresses human breast and lung cancer xenografts. Proc Natl Acad Sci U S A 109: 9623–9628. 22623533 10.1073/pnas.1121606109 PMC3386073

[30] Cohen EE, Lingen MW, Zhu B, Zhu H, Straza MW, et al (2006) Protein kinase C zeta mediates epidermal growth factor-induced growth of head and neck tumor cells by regulating mitogen-activated protein kinase. Cancer Res 66: 6296–6303. 16778206 10.1158/0008-5472.CAN-05-3139

[31] Valkova C, Mertens C, Weisheit S, Imhof D, Liebmann C (2010) Activation by tyrosine phosphorylation as a prerequisite for protein kinase Czeta to mediate epidermal growth factor receptor signaling to ERK. Mol Cancer Res 8: 783–797. 20407013 10.1158/1541-7786.MCR-09-0164

[32] Xiao H, Bai XH, Wang Y, Kim H, Mak AS, et al (2013) MEK/ERK pathway mediates PKC activation-induced recruitment of PKCzeta and MMP-9 to podosomes. J Cell Physiol 228: 416–427. 22740332 10.1002/jcp.24146

[33] Fernandez N, Caloca MJ, Prendergast GV, Meinkoth JL, Kazanietz MG (2000) Atypical protein kinase C-zeta stimulates thyrotropin-independent proliferation in rat thyroid cells. Endocrinology 141: 146–152. 10614633 10.1210/endo.141.1.7278

[34] Zhang X, Yue P, Fletcher S, Zhao W, Gunning PT, et al (2010) A novel small-molecule disrupts Stat3 SH2 domain-phosphotyrosine interactions and Stat3-dependent tumor processes. Biochem Pharmacol 79: 1398–1409. 20067773 10.1016/j.bcp.2010.01.001 PMC3188443

[35] Luna-Ulloa LB, Hernandez-Maqueda JG, Santoyo-Ramos P, Castaneda-Patlan MC, Robles-Flores M (2011) Protein kinase C zeta is a positive modulator of canonical Wnt signaling pathway in tumoral colon cell lines. Carcinogenesis 32: 1615–1624. 21859831 10.1093/carcin/bgr190

[36] Yao S, Bee A, Brewer D, Dodson A, Beesley C, et al (2010) PRKC-zeta Expression Promotes the Aggressive Phenotype of Human Prostate Cancer Cells and Is a Novel Target for Therapeutic Intervention. Genes Cancer 1: 444–464. 21779455 10.1177/1947601910376079 PMC3092210

[37] Guo H, Gu F, Li W, Zhang B, Niu R, et al (2009) Reduction of protein kinase C zeta inhibits migration and invasion of human glioblastoma cells. J Neurochem 109: 203–213. 19187446 10.1111/j.1471-4159.2009.05946.x

[38] Huang S, Ouyang N, Lin L, Chen L, Wu W, et al (2012) HGF-induced PKCzeta activation increases functional CXCR4 expression in human breast cancer cells. PLoS ONE 7: e29124. 22242160 10.1371/journal.pone.0029124 PMC3252308

[39] Valkov A, Sorbye SW, Kilvaer TK, Donnem T, Smeland E, et al (2011) The prognostic impact of TGF-beta1, fascin, NF-kappaB and PKC-zeta expression in soft tissue sarcomas. PLoS ONE 6: e17507. 21390241 10.1371/journal.pone.0017507 PMC3048407

[40] Nakanishi H, Brewer KA, Exton JH (1993) Activation of the zeta isozyme of protein kinase C by phosphatidylinositol 3,4,5-trisphosphate. J Biol Chem 268: 13–16. 8380153

[41] Le Good JA, Ziegler WH, Parekh DB, Alessi DR, Cohen P, et al (1998) Protein kinase C isotypes controlled by phosphoinositide 3-kinase through the protein kinase PDK1. Science 281: 2042–2045. 9748166 10.1126/science.281.5385.2042

[42] Chou MM, Hou W, Johnson J, Graham LK, Lee MH, et al (1998) Regulation of protein kinase C zeta by PI 3-kinase and PDK-1. Curr Biol 8: 1069–1077. 9768361 10.1016/s0960-9822(98)70444-0

[43] Scholz A, Heinze S, Detjen KM, Peters M, Welzel M, et al (2003) Activated signal transducer and activator of transcription 3 (STAT3) supports the malignant phenotype of human pancreatic cancer. Gastroenterology 125: 891–905. 12949733 10.1016/s0016-5085(03)01064-3

[44] DeArmond D, Brattain MG, Jessup JM, Kreisberg J, Malik S, et al (2003) Autocrine-mediated ErbB-2 kinase activation of STAT3 is required for growth factor independence of pancreatic cancer cell lines. Oncogene 22: 7781–7795. 14586404 10.1038/sj.onc.1206966

[45] Fukuda A, Wang SC, Morris JPt, Folias AE, Liou A, et al (2011) Stat3 and MMP7 contribute to pancreatic ductal adenocarcinoma initiation and progression. Cancer Cell 19: 441–455. 21481787 10.1016/j.ccr.2011.03.002 PMC3075548

[46] Jaganathan S, Yue P, Turkson J (2010) Enhanced sensitivity of pancreatic cancer cells to concurrent inhibition of aberrant signal transducer and activator of transcription 3 and epidermal growth factor receptor or Src. J Pharmacol Exp Ther 333: 373–381. 20100905 10.1124/jpet.109.162669 PMC2872953

[47] Nam S, Wen W, Schroeder A, Herrmann A, Yu H, et al (2012) Dual inhibition of Janus and Src family kinases by novel indirubin derivative blocks constitutively-activated Stat3 signaling associated with apoptosis of human pancreatic cancer cells. Molecular oncology 10.1016/j.molonc.2012.10.013 PMC3968804 23206899

[48] Yue P, Turkson J (2009) Targeting STAT3 in cancer: how successful are we? Expert Opin Investig Drugs 18: 45–56. 10.1517/13543780802565791 PMC2610472 19053881

[49] Frohner W, Lopez-Garcia LA, Neimanis S, Weber N, Navratil J, et al (2011) 4-benzimidazolyl-3-phenylbutanoic acids as novel PIF-pocket-targeting allosteric inhibitors of protein kinase PKCzeta. J Med Chem 54: 6714–6723. 21863889 10.1021/jm2005892

[50] Lopez-Garcia LA, Schulze JO, Frohner W, Zhang H, Suss E, et al (2011) Allosteric regulation of protein kinase PKCzeta by the N-terminal C1 domain and small compounds to the PIF-pocket. Chemistry & Biology 18: 1463–1473. 22118680 10.1016/j.chembiol.2011.08.010

