# Supplementary figures and images for: Protein Kinase C Zeta Regulates Human Pancreatic Cancer Cell Transformed Growth and Invasion through a STAT3-Dependent Mechanism
Source: PLoS One. 2013 Aug 28;8(8):e72061. doi: 10.1371/journal.pone.0072061 (PMC3756013; doi:10.1371/journal.pone.0072061)

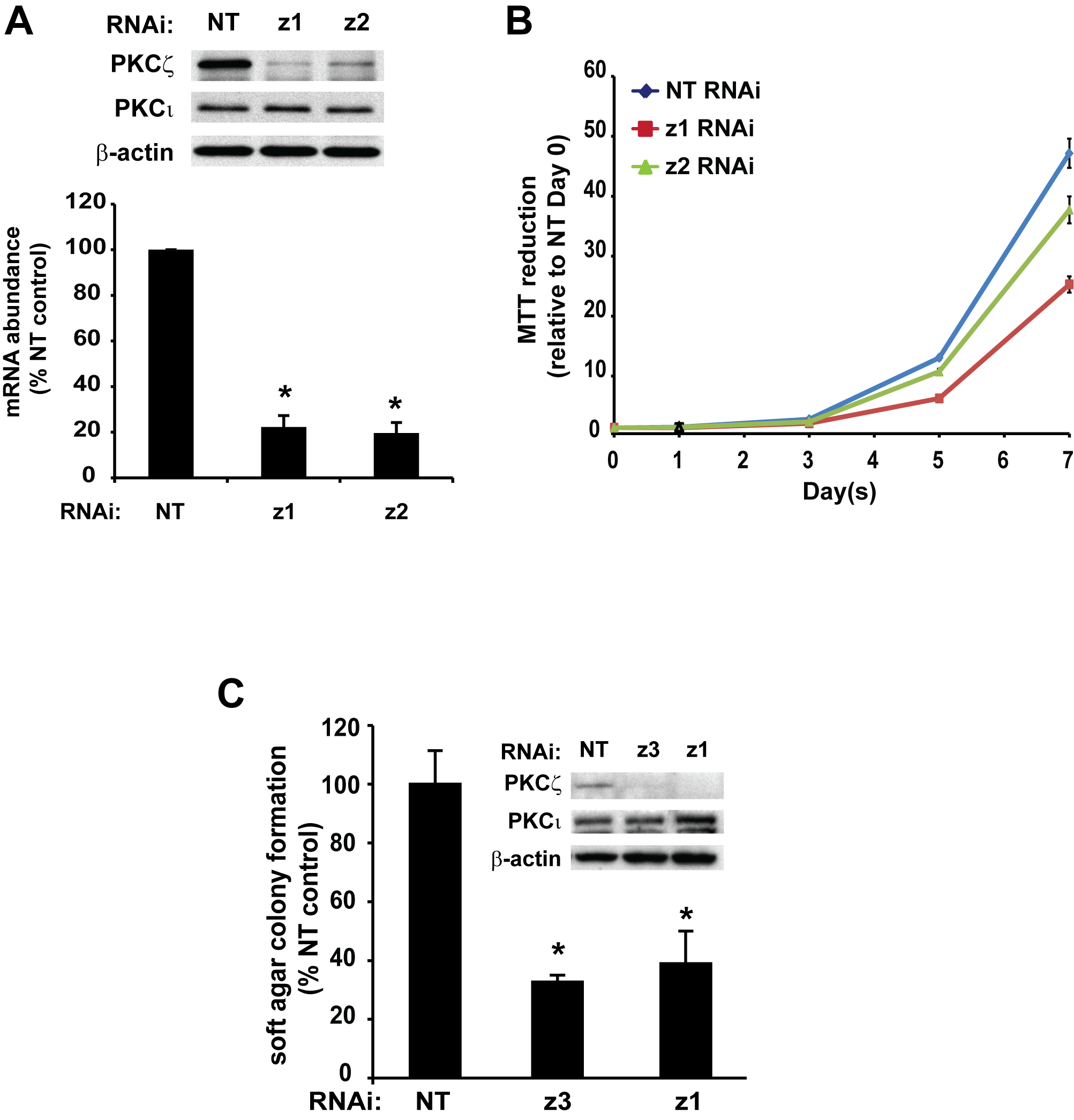

Supplement: Figure S1 — Inhibition of PKCζ expression reduces survival and transformed growth of MiaPaca-2 pancreatic cancer cells. MiaPaca-2 cells stably carrying lentiviral constructs expressing either control, non-targeting (NT), or PKCζ-targeting RNAi (z1 and z2) were assessed for A) PKCζand PKCι protein expression by immunoblot analysis (top), and PKCζ mRNA abundance by qPCR analysis (bottom); B) cell viability (MTT colorimetric assay); C) anchorage-independent growth (colony formation in soft agar). PKCζ RNAi #3 (z3) construct targets a sequence in the coding region of PKCζ (CATGAAAGTGGTGAAGAAAGA). For each panel Bars = average of 3 or more replicates±SD and graph is representative of 2 or more independent experiments. *p<0.05 vs NT. (TIF) [file pone.0072061.s001.tif]

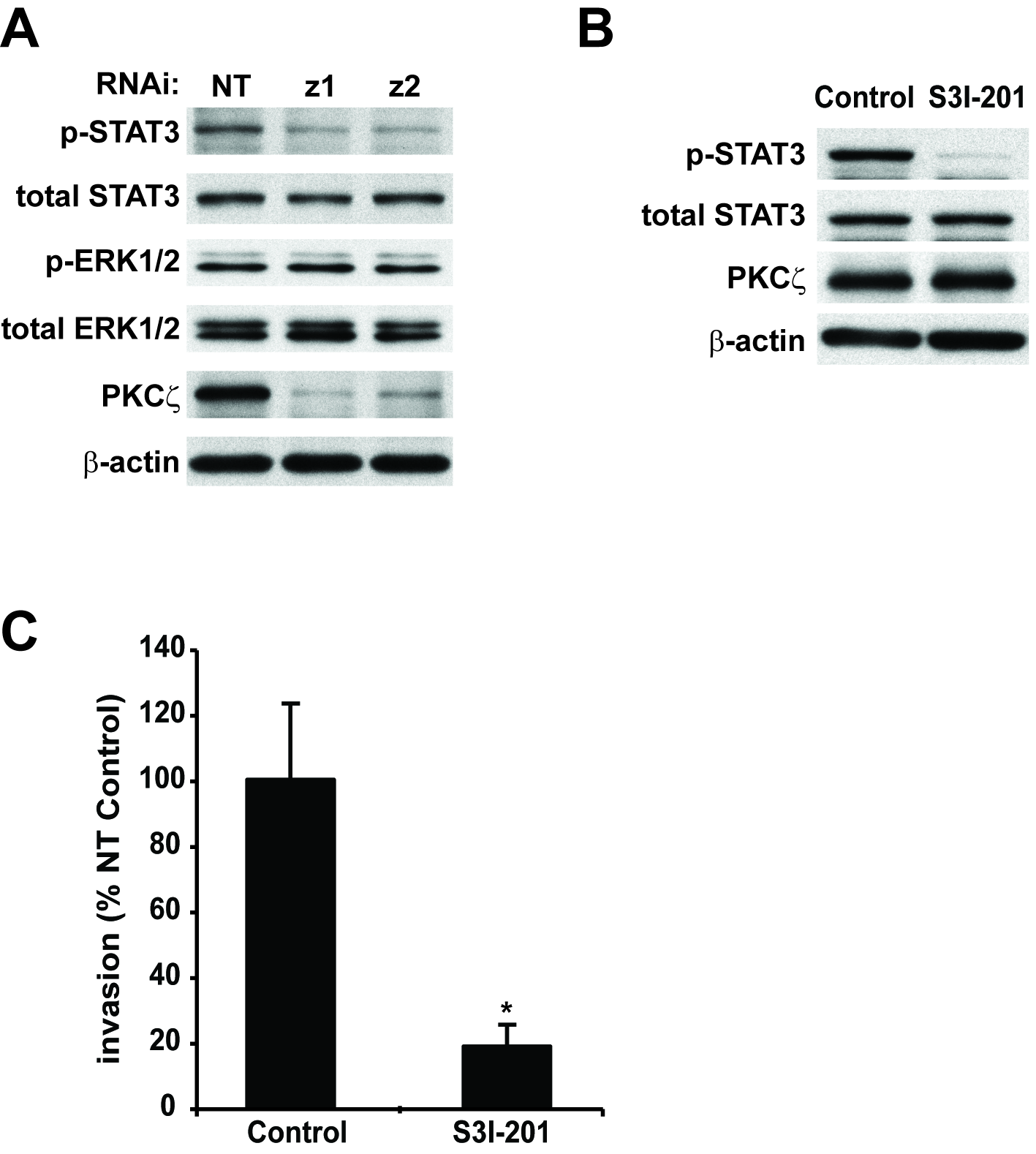

Supplement: Figure S3 — PKCζ expression regulates STAT3 activation in MiaPaca-2 cells. A) Inhibition of PKCζ expression decreases constitutive STAT3 activation (p-STAT3) but has no effect on ERK1/2 phosphorylation (p-ERK). Immunoblot analysis was performed on total cell lysates from MiaPaca-2 NT and PKCζ RNAi cells. B) Inhibition of STAT3 (S3I-201) decreases p-STAT3. Immunoblot analysis was performed on total cell lysates from MiaPaca-2 NT and PKCζ RNAi cells. C) S3I-201 significantly reduces MiaPaca-2 cell invasion. Bars = average of 3 or more replicates±SD and graph is representative of 2 or more independent experiments. *p<0.05 vs Control. For all panels S3I-201 was used at 100 µm with DMSO as control diluent. (TIF) [file pone.0072061.s003.tif]
